# Comparison of rapid and conventional methods for investigating of mecA presence in Staphylococcus Species

**DOI:** 10.12669/pjms.40.4.9284

**Published:** 2024

**Authors:** Mehmet Hakan Taskin

**Affiliations:** Department of Medical Microbiology, Samsun University, Faculty of Medicine, Samsun, Türkiye. December 14, 2023 mhtaskin@gmail.com

This refers to the above mentioned manuscript published in Pakistan Journal of Medical Science 2021; 37(5) 1467-1474.^1^ It aimed to compare the reliability levels of disc diffusion, latex agglutination test and chromogenic agar methods by referring to the detection of mecA gene by polymerized chain reaction (PCR) in the determination of methicillin resistance in Staphylococcus species.

In this article, the detection of the mentioned gene region by PCR is referenced in the method (MecA PCR: The multiplex PCR protocol used in the study was as follows: 10x PCR Buffer 2.5 µL, 10 mM dNTP 0.5 µL, MECA 1 (10 pmol) 1.25 µL, MECA 2 (10 pmol) 1.25 µL, 25 mM MgCl23 µL, DNA Polymerase 0.5 µL, distilled water 13.5 µL and bacterial DNA 2.5 µL. Thermal Cycler phase: 1 cycle at 94o C is 2 minutes, at 94o C 35 cycles 15 sec, at 55o C 35 cycles 30 seconds, at 72o C 35 cycles 30 seconds and at 72o C 1 cycle 10 minutes.) MecA PCR is shown as multiplex PCR. Although this expression is used for multiple gene regions, it is not clear for which gene regions it is written here. It is also not clear what is meant by MECA1 and MECA2 expressions mentioned in the PCR protocol. It is also not clear at what volts per cm2 in what percentage agarose the electrophoresis was performed. These features are important for the reliability of the study. It is not clear how the results were determined and interpreted in this article, where neither the source nor the primer sequences nor the target site for the primers are specified. The phrase “MgCl23” in the parenthetical protocol section is not understood. In other words, I think that the contents of the protocol and method are not clear. These parts and their effects on the results should be documented.

**REFERENCE**

1. Gorgun S, Isler H, Turgut MC. Comparison of rapid and conventional methods for investigating of mecA presence in Staphylococcus Species. Pak J Med Sci. 2021;37(5):1467-1474. doi: 10.12669/pjms.37.5.4274

## Response from the authors:

We have read with great interest the comments by the reader on our study. We are grateful to him for the interest shown in our manuscript.

The reader has stated that the MECA-1 and MECA-2 expressions and the target region were not specified in our study, that the running voltage used in electrophoresis was not included in the article, that they did not understand what MgCl23 in the method meant, and that it was not clear how our findings were interpreted.

First of all, the expression “MgCl23” is mentioned as “25 mM MgCl23 μL” in the article, and it should not be difficult to understand that this is 3 μL of MgCl2 used in PCR.

In our study, it was stated that the sensitivity and specificity between the methods were compared in terms of the presence of the mecA gene. It was also stated that this was done as follows:

“Sensitivity, specificity, positive predictive value, negative predictive value, and accuracy of the methods were calculated. For sensitivity, the ratio of the number of isolates found to be resistant to methicillin to the actual number of resistant isolates, and for specificity, the ratio of the number of isolates found to be susceptible to methicillin to the actual number of susceptible isolates was calculated. The positive predictive value was calculated from the ratio of the true resistant isolates among the isolates found to be resistant to methicillin, and the negative predictive value from the ratio of the actual susceptible isolates among the isolates found to be susceptible to methicillin. Finally, accuracy was calculated as the ratio of the total number of methicillin-resistant and susceptible isolates determined by the method to the total number of isolates.” In other words, the main subject of the study was a comparison between the methods in this respect, and even how sensitivity and specificity are calculated, which is rarely explained in the articles, is included in detail, which is not even necessary. What sensitivity is one of the most basic academic knowledge? However, the reader for some reason, did not find even this very detailed information sufficient.

The mecA gene in staphylococci was first discovered by Murakami et al.^1^ Later studies used the same method. As seen in their articles, these researchers shared the imaging findings as a result of PCR and showed the 533 bp mecA band. In the images in our study, the same band is seen as 533 bp and is clearly stated in the image footnote. In our study, the 5’ AAAATCGATGGTAAAGGTTGGC primer (forward) targeting the 1282 - 1303 region used by Murakami et al. and the 5’ AGTTCTGCAGTACCGGATTTGC primer (reverse) targeting the 1793 – 1814 region were used. In order to amplify the targeted gene region in PCR processes, it is necessary to use two primers, called forward and reverse, belonging to the beginning and ending parts of that region, and that the distance between the first point and the end point represents the size of the band sought (here 1824-1282 = 533 bp) when PCR was first discovered. It is one of the most basic and simple information known since day one. Therefore, it is surprising that the worthy reader did not understand that the names MECA1 and MECA2 in our study were forward and reverse primers.

The reader has also commented that the voltage used in electrophoresis was not specified. Murakami et al.^1^ also did not specify voltage information in their reference methods for mecA detection! So, aren’t Murakami et al.’s findings “reliable”? The voltage used in gel electrophoresis and the running time during electrophoresis only play a role in whether sufficient separation of the bands is achieved and whether the target band can be clearly seen in the imaging to be performed. The voltage used affects the size of the target band.^2^-^5^ Whether these factors work or not is evident from the imaging results. The quality of the band images in the gel run in our study is at a very high level. In other words, the voltage used was applied quite appropriately. Still, it is scientifically strange that the reader thinks that the voltage issue affects the reliability of the research. It is also surprising that the reader has even questioned marks regarding the voltage in electrophoresis.

As a result, in our study, the exact same reference method was applied from beginning to end, the gene region bands obtained were displayed in high quality and shared in the article, and even how the findings were interpreted was clearly explained down to the smallest detail. All this shows that our study data is extremely reliable.
